# An infant with Joubert syndrome: A case report

**DOI:** 10.1016/j.radcr.2022.11.024

**Published:** 2022-12-05

**Authors:** Shehroze Tabassum, Aroma Naeem, Rana Uzair Ahmad, Farhan Naeem, Faiza Afzal

**Affiliations:** King Edward Medical University, Nila Gumbad Chawk, Lahore, Punjab, 54000, Pakistan

**Keywords:** Joubert syndrome, Developmental milestones, Neuropathological abnormalities

## Abstract

Joubert syndrome is a rare neurological and developmental malfunction represented by decreased muscle tone, ataxia, and delayed developmental milestones. Joubert syndrome-related disorders, besides central nervous system, can involve other systems and thus can lead to multi-organ malfunction. We report a case of pure Joubert syndrome who presented with developmental delay, decreased muscle tone, and ataxia. Identification of molar tooth sign on magnetic resonance imaging studies assisted to make a definitive diagnosis. Detailed examination revealed no other significant findings of any organ of the body. Patient was managed conservatively with symptomatic treatment. Although these types of cases are rarely encountered, they can lead to multiple organ disabilities. Therefore, clinicians should always keep this diagnosis in mind whenever an infant presents with the aforementioned neurodevelopmental symptoms.

## Introduction

Joubert syndrome (JS) is an uncommon autosomal recessive disease described by abnormal breathing patterns, developmental retardation, ataxia, eye movements, hypotonia coupled with neuropathological abnormalities of brainstem and cerebellum such as aplasia of vermis and inherited hypoplasia. In 1969, this disease was named after Marie Joubert. It has a prevalence of less than 1 in 100,000, making it very rare [1,2]. A triad of developmental delays, hypotonia, and characteristic molar tooth sign (MTS) on magnetic resonance imaging studies (MRI) of brain define the classic form of JS [2,3]. Recently, a new term, Joubert syndrome-related disorders (JSRD) has been tossed to include neurological pathologies, having features of MTS coupled with the involvement of other organs. Six clinical phenotypes have been discovered on the basis of involvement of other organs [2,4]. Since JS is considered as a syndrome with different phenotypes and the average age of diagnosis is 33 months, therefore, it is difficult to diagnose the exact subtypes of JS in a newborn child [5]. Pattern of JS can be sporadic or autosomal recessive [6]. We present a case of a 9-month old infant with classic features of JS including delayed developmental milestones, ataxia, and molar tooth sign on MRI. This is a rarely reported syndrome and is difficult to diagnose without imaging studies. Even though MTS is not always a positive finding in all patients with JS, MRI is still the initial test of choice. In order to avoid any significant fatal abnormalities, pediatricians and neurologists should collaborate to diagnose and manage this disorder in the early stages.

## Case presentation

**Fig. 1.1:**
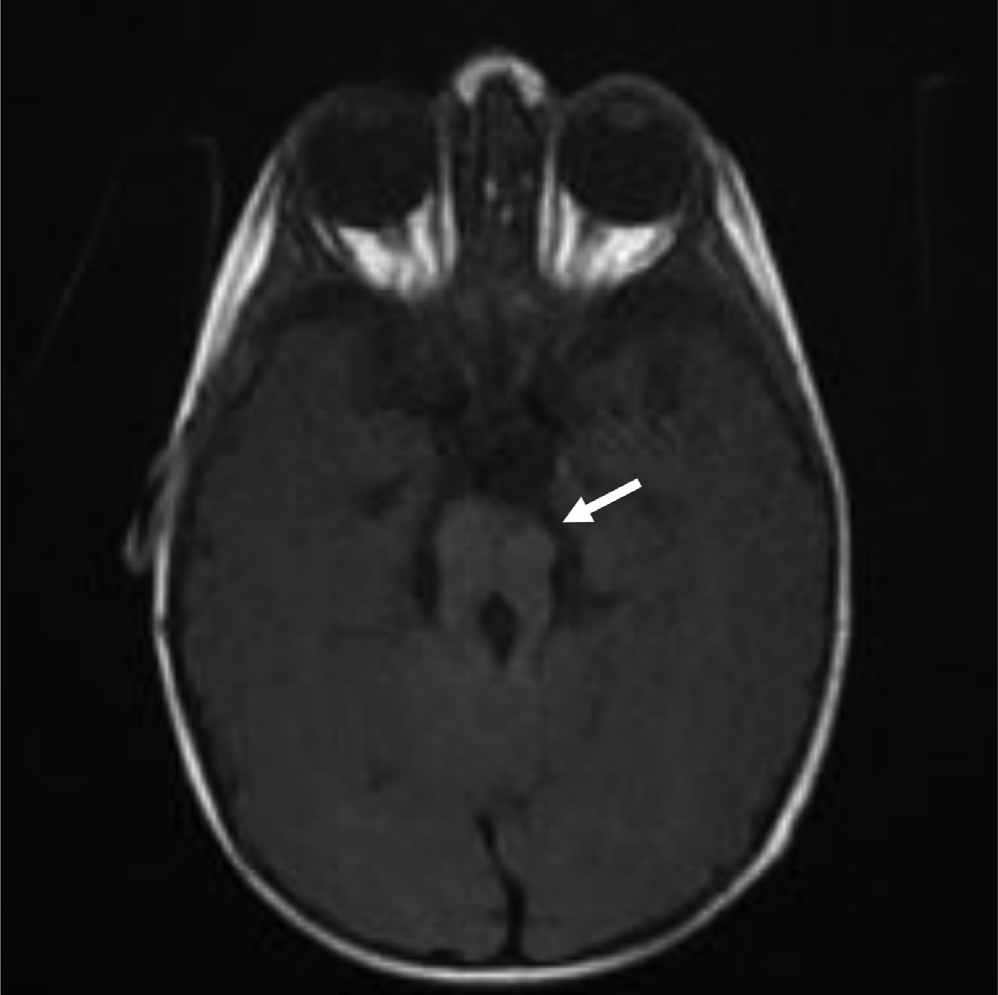
T1WI sequence—axial section.

**Fig. 1.2:**
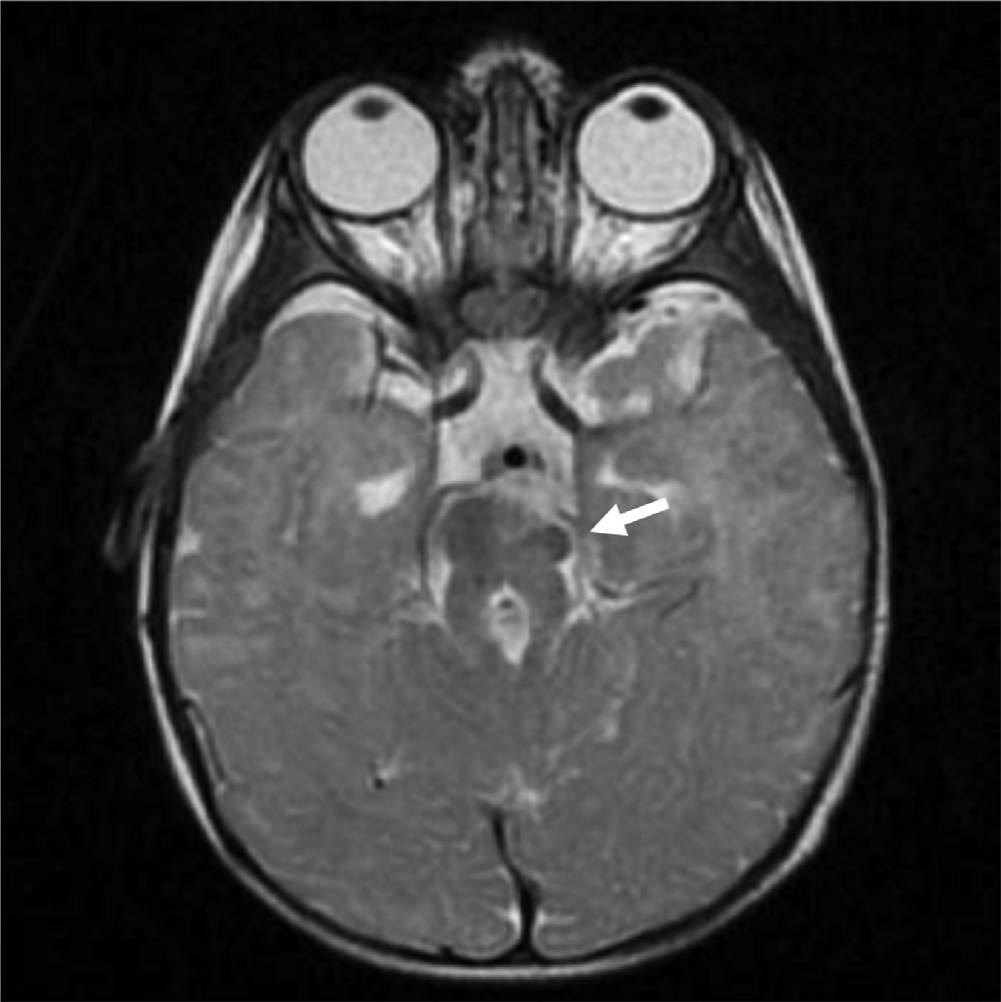
T2WI sequence—axial section.

**Fig. 1.3:**
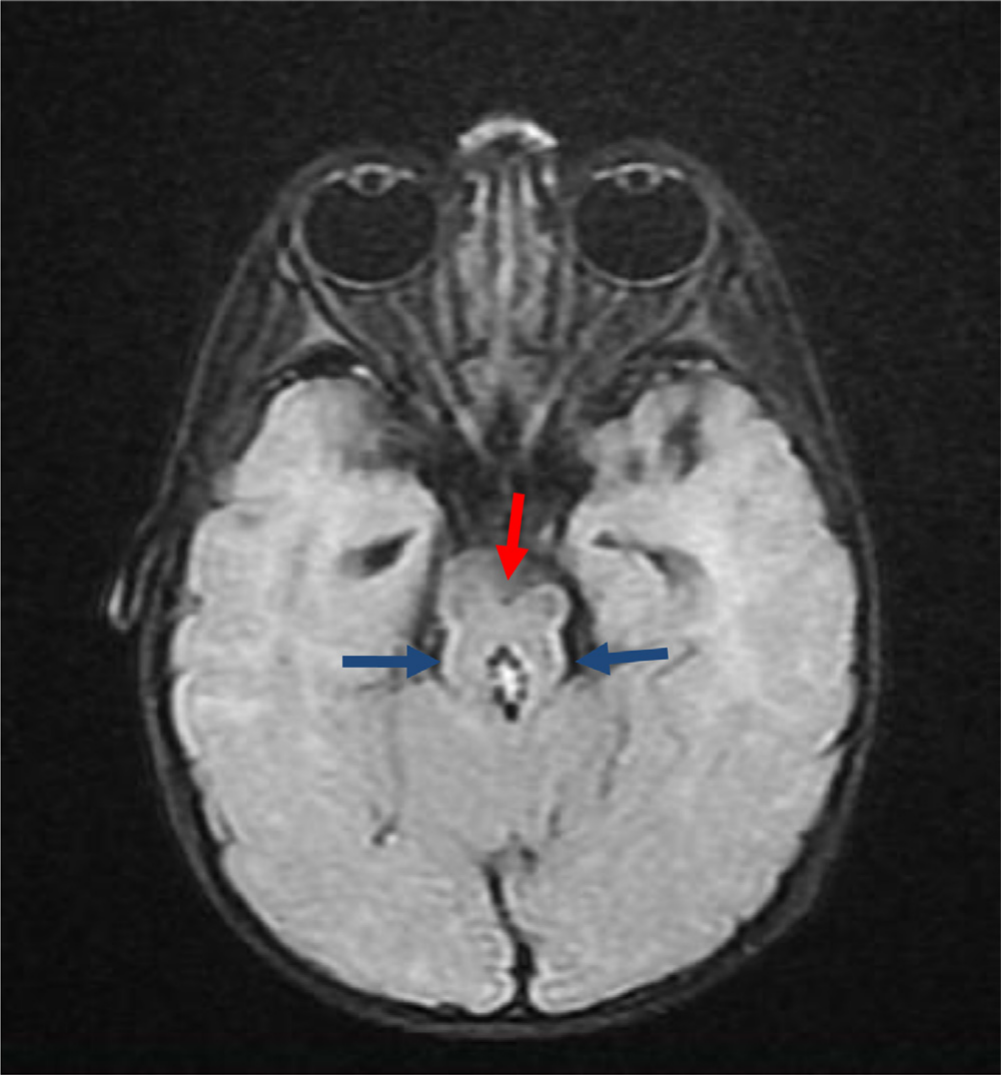
FLAIR sequence—axial section. T1-weighted image (T1WI), T2-weighted image (T2WI), and fluid-attenuated inversion recovery (FLAIR) sequences showing elongated and thickened superior cerebellar peduncles (marked by blue arrow) with hypoplasia of isthmus and deep cleft along anterior midbrain (marked by red arrow) giving characteristic molar tooth sign as marked by white arrows. Interpeduncular fossa is also widened. There is associated hypoplasia of cerebellar vermis.

**Fig. 2:**
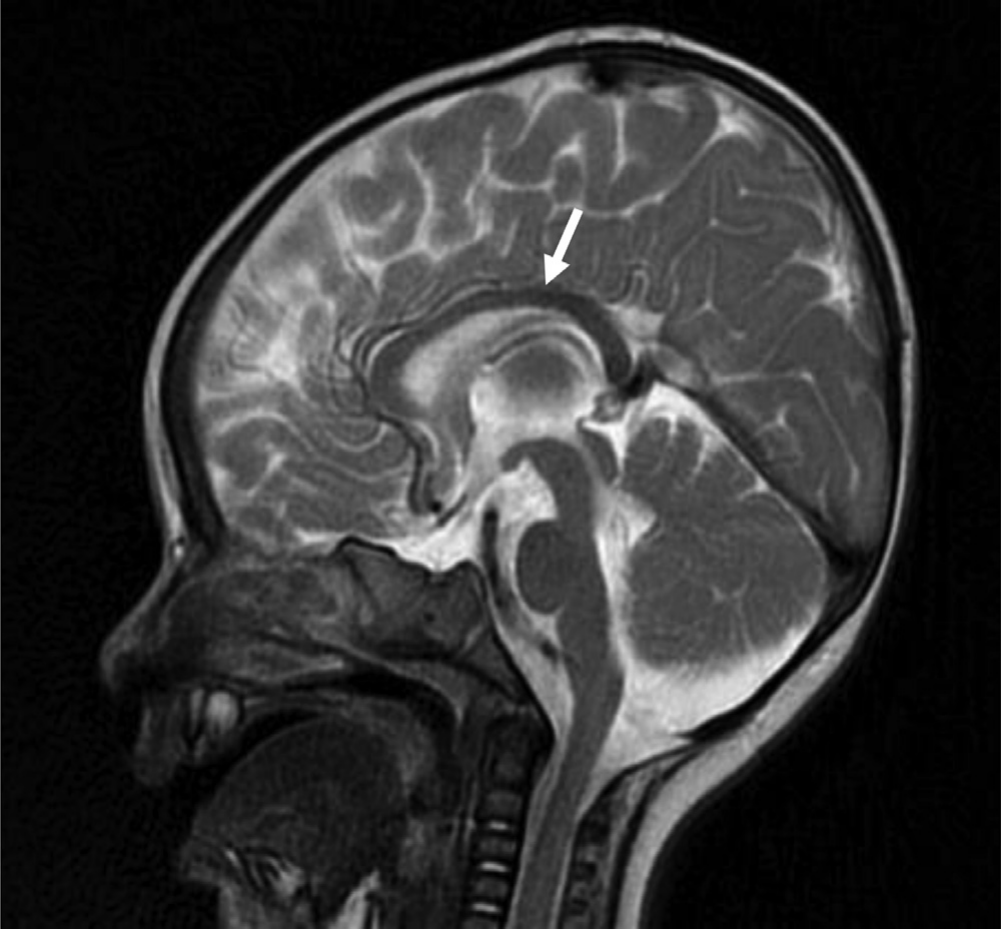
T2WI sequence—mid sagittal section. T2WI sequence, mid sagittal section showing hypoplasia of corpus callosum marked by white arrow, a feature associated with Joubert syndrome. Vermian hypoplasia is also appreciated.

A 9-month-old boy presented through outpatient department (OPD) of our hospital with delayed developmental milestones, hypotonia, and ataxia. Patient achieved social smile at the age of 2-and-half months and was still unable to hold his neck at the age of 9 months. Pregnancy was uneventful with normal spontaneous vaginal delivery without any history of birth asphyxia. His parents had consanguine marriage. No respiratory or oculomotor abnormalities were observed during examination. MRI of the brain was advised for the patient and sent to radiology department. Findings shown by the MRI of the patient are shown and described in Fig. 1.1, Fig. 1.2, Fig. 1.3 and 2.

All these findings of imaging studies and presenting symptoms led to the diagnosis of pure JS due to the absence of multi-organ involvement. Patient was managed conservatively and his parents were counseled regarding the syndrome.

## Discussion

JS is a rare brain malformation characterized by total or partial vermian hypoplasia, molar tooth sign on imaging studies and a wide spectrum of symptoms like hyperpnoea, decreased muscle tone, neurological abnormalities, oculomotor apraxia, incoordination of voluntary muscles, and delayed developmental milestones. The first ever case of the syndrome was reported in 1968 with characteristic multiple neurological abnormalities and episodes of increased respiratory rate [2]. It is a rarely reported disorder. Until 2009, the overall number of cases reported in the literature approximate to 200 [7]. Although JS patients have no associated evident facial appearance, not even seen in our case but many patients have shown distinguishing facial features, like prominent lower jaw which can be a supportive finding in helping with the diagnosis [8,9]. Besides the clinical diagnosis, studies that deal with genotype-phenotype correlation can provide important clues about the risk of recurrence as well as help in predicting occurrence of disease in successive generations [10], [11], [12], [13]. Disease with autosomal recessive pattern is related to mutations in various genes like NPHP1, CEP290, and AHI1 [6]. These mutations affect multiple signaling pathways resulting in irregular proliferation and migration of neuronal cells, ultimately leading to various neurological and respiratory abnormalities [2]. JS includes only those patients who present with delayed developmental milestones, molar tooth sign, and batwing appearance of fourth ventricle, brainstem and cerebellar vermis abnormalities. However, patients with JSRD present with clinical and imaging manifestations of JS as well as multiple other symptoms related to other organs of the body. Although central nervous system (CNS) is the primary system involved, extra CNS involvement usually includes the eyes, esophagus, kidney, liver, spleen, and any part of the gastrointestinal tract [4,14]. Due to multi-organ involvement, referral to subspecialties at the time of presentation can aid in early diagnosis and improved quality of life. Every organ in the body should be tested thoroughly in order to subsequently aid in clear identification of the spectrum of the disorder. There is no definitive treatment available for the disease, so patients are managed conservatively with symptomatic treatment involving a combination of cognitive, behavioral, occupational, and psychiatric strategies in order to manage the wide spectrum of disease [7]. Special language classes for speaking, special schooling for appropriate skills, and annual screening for the disease are recommended for every patient with JS [15]. Our patient presented with hypotonia and clumsy voluntary movements. After thorough examination of all the organs and systems of the body, and finding a characteristic molar tooth sign on MRI, the patient was labeled as a case of pure JS. These types of cases are extremely rare throughout the world, difficult to diagnose and usually lead to a wide range of disabilities due to possibilities of late diagnosis. Adding to this, most of the patients lack ease of access to subspecialists (neurologists, psychiatrists, pediatricians, etc.) who must be consulted in order to promptly diagnose and treat the disease. This highlights the need to instill awareness among the affected individuals about the importance of referral to subspecialties in order to improve quality of care among patients with JS. Clinicians should keep this diagnosis in mind whenever any infant presents with the above mentioned symptoms and imaging studies should be consulted as early as possible to help in early diagnosis and prompt management of patients with JS.

## Conclusion

Joubert syndrome is an autosomal recessive disorder that usually presents as delay in developmental milestones, decreased tone of the muscles, abnormalities in the cerebellum and brainstem. The disease being extremely rare is often missed at the time of presentation. An effective way of improving the quality of care in Joubert syndrome patients involves timely referral of such patients to subspecialists in order to exclude multi-organ involvement. Such efforts can be aided by evolvement of gene targeting treatments that can facilitate in provision of precise treatment and thus a better quality of life to patients suffering from this syndrome.

## Ethical approval

Not required as we have acquired consent from the patient.

## Author contribution

All authors contributed equally.

## Patient consent

Written informed consent was obtained from the mother of patient for publication of this case report and accompanying images. A copy of the written consent is available for review by the Editor-in-Chief of this journal on request.

## Research registration

1. Name of the registry: NA
2. Unique Identifying number or registration ID: NA
3. Hyperlink to your specific registration (must be publicly accessible and will be checked): NA

## Provenance and peer review

Not commissioned, externally peer reviewed.

## References

[1] Choh SA, Choh NA, Bhat SA, Jehangir M. (2009). MRI findings in Joubert syndrome. Indian J Pediatr.

[2] Brancati F, Dallapiccola B, Valente EM. (2010). Joubert syndrome and related disorders. Orphanet J Rare Dis.

[3] Parisi MA. (2009). Clinical and molecular features of Joubert syndrome and related disorders. Am J Med Genet C Semin Med Genet.

[4] Elhassanien AF, Alghaiaty HAA. (2013). Joubert syndrome: clinical and radiological characteristics of nine patients. Ann Indian Acad Neurol.

[5] Singh P, Goraya JS, Saggar K, Ahluwalia A. (2011). A report of Joubert syndrome in an infant, with literature review. J Pediatr Neurosci.

[6] Valente EM, Dallapiccola B, Bertini E. (2013). Joubert syndrome and related disorders. Handb Clin Neurol.

[7] Bin Dahman HA, Bin Mubaireek AHM, Alhaddad ZH (2016). Joubert syndrome in a neonate: case report with literature review. Sudan J Paediatr.

[8] Braddock SR, Henley KM, Maria BL. (2007). The face of Joubert syndrome: a study of dysmorphology and anthropometry. Am J Med Genet A.

[9] Maria BL, Boltshauser E, Palmer SC, Tran TX. (1999). Clinical features and revised diagnostic criteria in Joubert syndrome. J Child Neurol.

[10] Bachmann-Gagescu R, Dempsey JC, Phelps IG, O’Roak BJ, Knutzen DM, Rue TC (2015). Joubert syndrome: a model for untangling recessive disorders with extreme genetic heterogeneity. J Med Genet.

[11] Brancati F, Barrano G, Silhavy JL, Marsh SE, Travaglini L, Bielas SL (2007). CEP290 mutations are frequently identified in the oculo-renal form of Joubert syndrome-related disorders. Am J Hum Genet.

[12] Doherty D, Parisi MA, Finn LS, Gunay-Aygun M, Al-Mateen M, Bates D (2010). Mutations in 3 genes (MKS3, CC2D2A and RPGRIP1L) cause COACH syndrome (Joubert syndrome with congenital hepatic fibrosis). J Med Genet.

[13] Vilboux T, Doherty DA, Glass IA, Parisi MA, Phelps IG, Cullinane AR (2017). Molecular genetic findings and clinical correlations in 100 patients with Joubert syndrome and related disorders prospectively evaluated at a single center. Genet Med.

[14] İncecik F, Hergüner MÖ, Altunbaşak Ş, Gleeson JG. (2012). Joubert syndrome: report of 11 cases. Turk J Pediatr.

[15] Akcakus M, Gunes T, Kumandas S, Kurtoglu S, Coskun A. (2003). Joubert syndrome: report of a neonatal case. Paediatr Child Health.

